# Improving prescriber confidence in COX-2 inhibitor use for aspirin-exacerbated respiratory disease: impact of a standard operating procedure

**DOI:** 10.3389/falgy.2026.1854011

**Published:** 2026-06-22

**Authors:** Emily Hocknell, Peta-Lee Sacks, Ulka Vamadevan, Claire Hopkins

**Affiliations:** Ear, Nose and Throat Department, Guys and St Thomas Hospital NHS Trust, London, United Kingdom

**Keywords:** analgesia, asthma, cyclooxygenase 2 inhibitors, drug hypersensitivity, pain, postoperative

## Abstract

**Introduction:**

Aspirin-exacerbated respiratory disease (AERD) presents a challenge in perioperative pain management. Many prescribers are uncertain about COX-2 inhibitor safety, leading to inconsistent prescribing practices and suboptimal analgesia in this group of patients. The aim of this small, single-centre study was to assess healthcare practitioners' confidence in prescribing analgesia for AERD patients and evaluate the impact of a standard operating procedure (SOP) on prescribing practices.

**Material and methods:**

An initial survey assessed practitioners' confidence in prescribing analgesia, particularly COX-2 inhibitors, for patients with AERD. Based on these findings, a SOP including a test dose protocol, was developed to provide clear guidance on prescribing guidance. A follow-up survey was conducted after implementation to reassess practitioner confidence.

**Results:**

Forty-four practitioners completed the initial survey; Nineteen (43%) felt comfortable managing pain in AERD, however only six (13.6%) were confident prescribing a COX-2 inhibitor for these patients. Following SOP implementation, seventeen practitioners were re-audited; sixteen (94.1%) found the SOP helpful in their decision making, and fourteen (82.4%) felt confident prescribing COX-2 inhibitors, reflecting an absolute increase of 68.8 percentage points in prescriber confidence.

**Discussion:**

This project demonstrates low confidence among practitioners in prescribing COX-2 inhibitors for patients with AERD. SOP implementation was reported to be helpful in decision-making and was associated with improved self-reported confidence, however differences between pre- and post-SOP survey groups warrant cautious interpretation. Clear prescribing guidance, including a test dose protocol may help address knowledge gaps and optimise pain management in this challenging patient population. However, effects on prescribing behaviour and patient outcomes remain to be established. We hope this small single centre implementation project will encourage other centres to develop and evaluate similar SOPs more systematically.

## Introduction

Aspirin exacerbated respiratory disease (AERD) is an inflammatory disease of the upper and lower airways usually characterised by symptoms of chronic hypertrophic eosinophilic rhinosinusitis, nasal polyposis and asthma (1). It is triggered by the ingestion of aspirin or non-steroidal anti-inflammatory drugs (NSAIDs) which cause inhibition of cyclooxygenase (COX) enzymes (1, 2). The COX enzyme has two known isoforms COX-1 and COX-2. Pharmaceutical inhibition of COX-2 can help to relieve pain and inflammation; however non-selective COX inhibitors inhibit varying amounts of both isoforms (2). The mechanism of this hypersensitivity reaction in asthmatics is complex. Trials to assess the cross reactivity of COX 2 inhibitors in AERD patients have strongly supported the inhibition of COX 1 as the essential initiator of this inflammatory process (3–5). Meloxicam and Nimesulide are two other types of medication that preferably inhibit COX 2 and therefore have a low prevalence of cross-reactivity in AERD. However, at higher doses can inhibit COX 1, eliciting mild respiratory reactions and therefore are best avoided in patients with AERD (5).

It is reported 5.4 million people in the UK suffer from asthma, with an estimated 5% to 15% of these patients having AERD (6, 7). In asthmatics with additional nasal polyposis the prevalence may be as high as 30% (7). The symptoms of AERD are difficult to treat, often not responding to standard medical interventions (1). Even with avoidance of NSAIDs and Aspirin, AERD patients will still usually experience progressive airway disease despite aggressive treatment (1). Surgical intervention for nasal polyps is often required, with a high rate of polyp recurrence and revision surgery (7). It is important to note, up to 15% of patients are also unaware they have NSAID hypersensitivity and will only be diagnosed via direct challenge (7).

Managing post operative pain in patients with AERD is challenging. Many healthcare professional feel uncomfortable prescribing COX-2 inhibitors in this population, despite evidence supporting their safety (3–5). Our project therefore does not aim to establish the safety of COX-2 inhibitors in AERD, but rather to address uncertainty around their use by implementing a structured SOP with a test dose protocol, and to evaluate its perceived impact on prescriber confidence.

## Materials and methods

An initial survey was created and distributed amongst ENT consultants, anaesthetists and pharmacists at a tertiary institution to assess clinicians' understanding and comfort in prescribing COX-2 inhibitors in AERD patients.

A standard operating procedure (SOP), including a test dose protocol, was then developed and implemented to guide the safe perioperative administration of COX-2 inhibitors in patients with AERD and support appropriate discharge prescribing of these medications once tolerance was confirmed.

**Figure 1:**
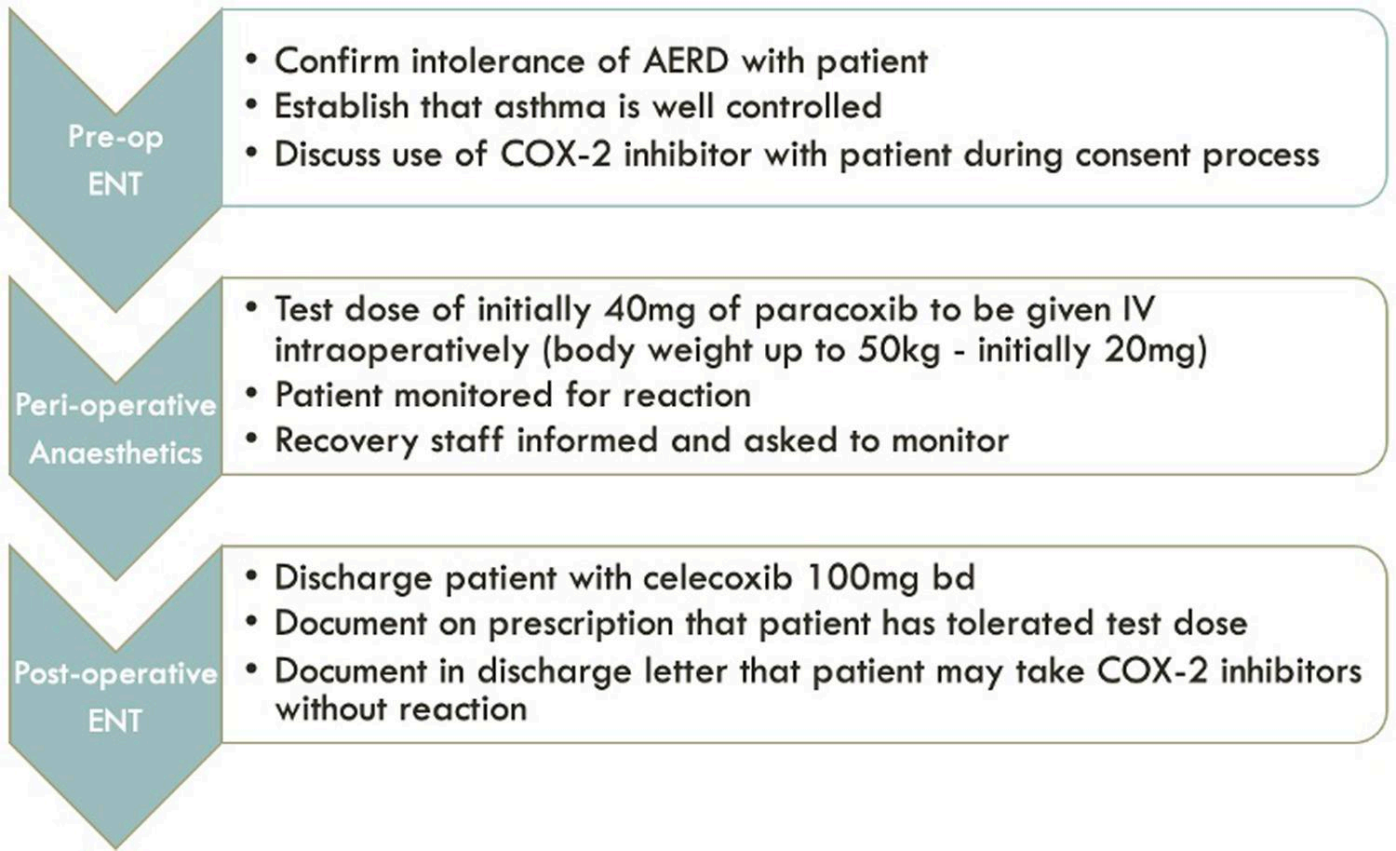
Quick reference guide for clinicians on how to administer COX-2 inhibitors with AERD.

The SOP only applied to adult patients (≥18 years) with AERD. It was intended for patients undergoing surgery in secondary or tertiary care settings. Paediatric patients were excluded from this protocol. The SOP outlined the scope of guidance for the use of COX-2 inhibitors in patients with AERD. It also included information on assessing asthma control, contraindications, and administering a supervised inpatient test dose of 40 mg intravenous (IV) parecoxib (20 mg if <50 kg) during surgical admission. If tolerated, these patients could be discharged with celecoxib 100 mg twice daily, with documentation confirming safe use. Figure 1 provides a quick reference guide included in the SOP.

Following the distribution of the SOP a follow up survey was conducted amongst the same group of prescribers, to reassess the understanding of prescribing COX-2 inhibitors in patients with AERD. Results were analysed using Fisher's exact test to compare the proportion of practitioners reporting comfort prescribing COX-2 inhibitors before and after SOP implementation.

## Results

### Baseline survey results

**Figure 2:**
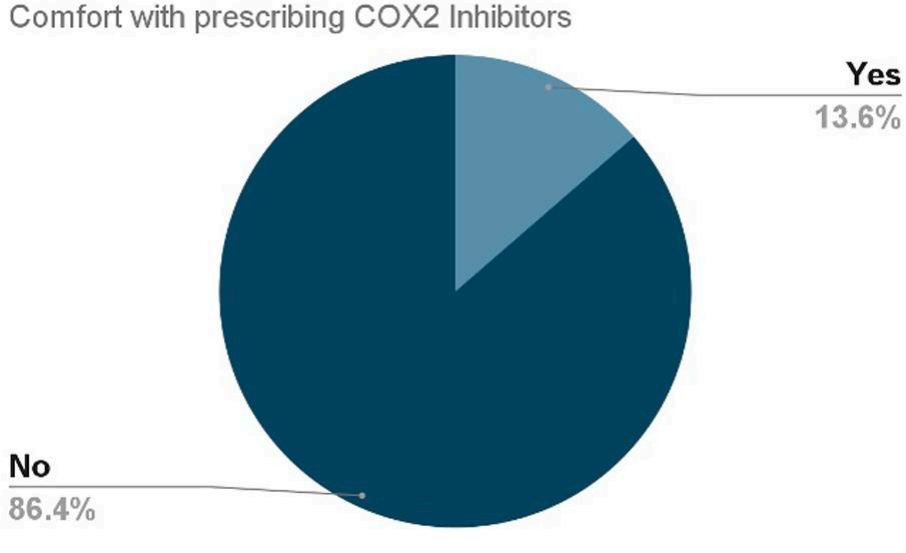
Distribution of practitioners who felt comfortable prescribing COX-2 inhibitors in patients with AERD at baseline prior to implementation of standard operating procedure.

Forty-four practitioners responded to the initial survey. Nineteen (43.2%) were Pharmacists, twenty-two (50%) were Anesthetists and three (6.8%) were ENT Consultants. Nineteen (43%) practitioners reported they initially felt comfortable managing pain relief in patients with AERD. When asked which types of analgesia they felt comfortable prescribing in patients with AERD, thirty-eight of forty-four respondents (86%) reported they would prescribe an opioid medication, however only six of forty-four respondents (13.6%) felt comfortable prescribe a COX-2 inhibitor (Figure 2).

### Post SOP survey results

Seventeen practitioners responded to the follow up survey after distribution of the SOP. There was a different proportion of professional groups compared to the pre-SOP survey, seven (41.2%) were ENT Consultants, eight (47.1%) were Anesthetists and two (11.8%) were Pharmacists. Sixteen (94.1%) practitioners reported they found the SOP helpful in their decision making.

**Figure 3:**
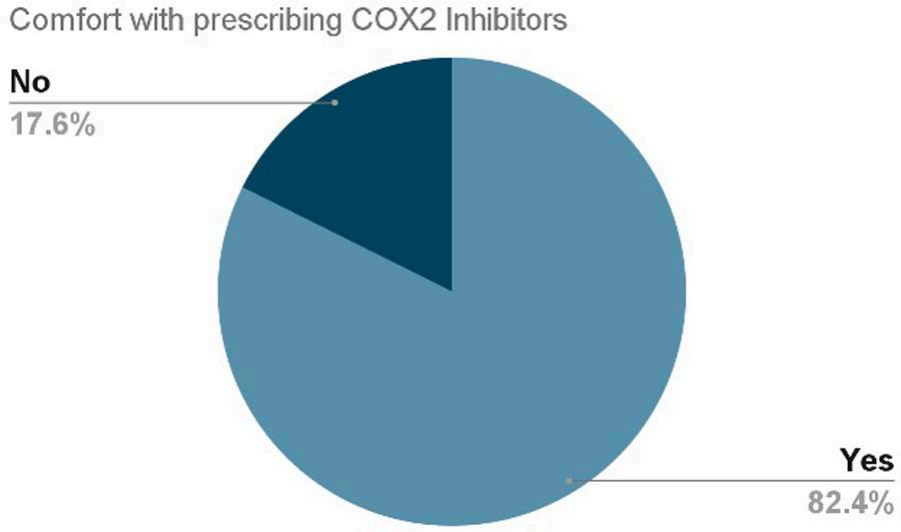
Distribution of practitioners who felt comfortable prescribing COX-2 inhibitors in patients with AERD following implementation of standard operating procedure.

**Figure 4:**
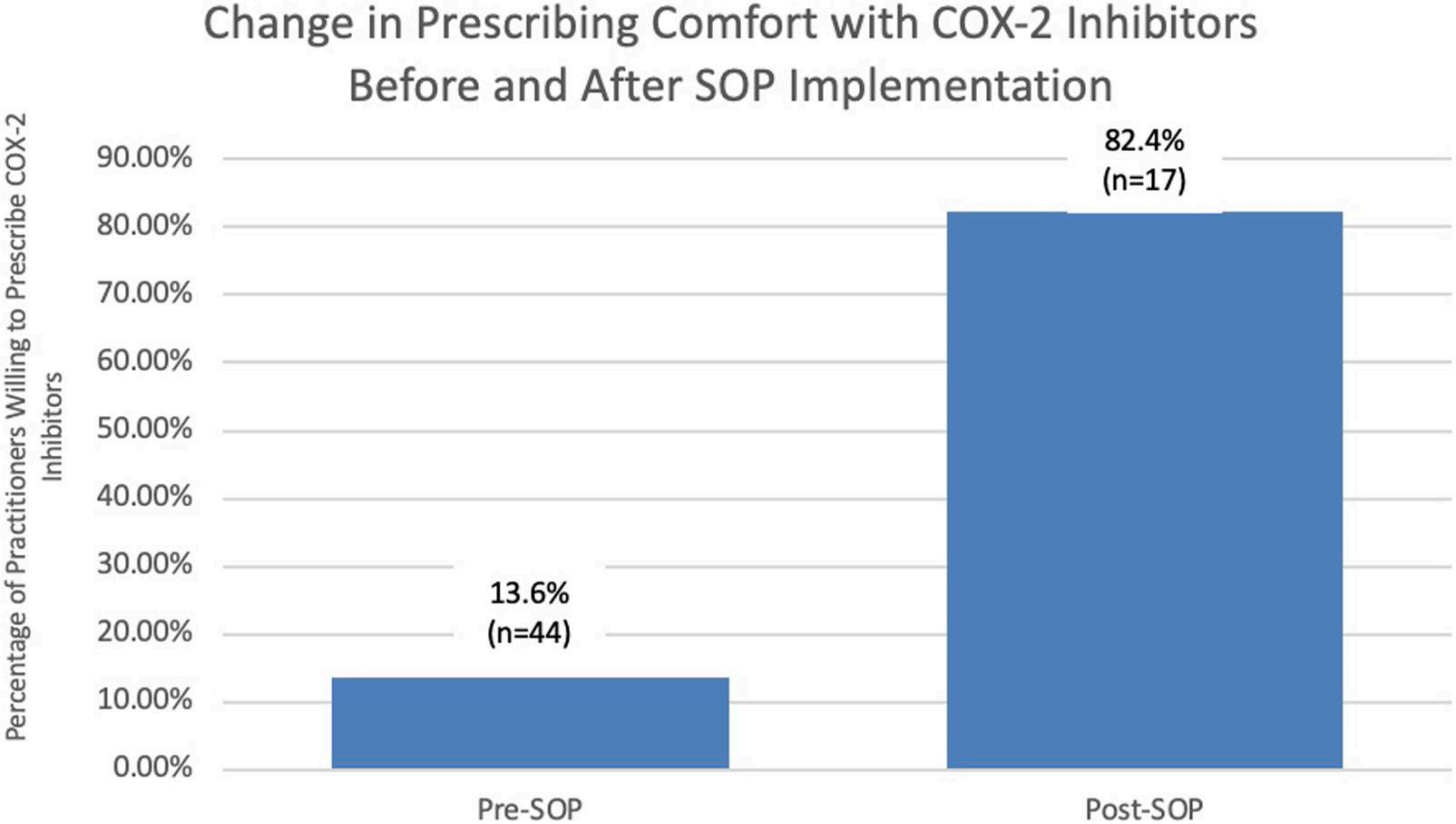
Change in prescribing comfort with COX-2 Inhibitors before and after SOP Implementation.

Following the distribution of our SOP, 14/17 (82.4%) practitioners reported feeling comfortable prescribing COX-2 inhibitors for patients with AERD (Figure 3), compared to 6/44 (13.6%) at baseline. This reflects an absolute increase of 68.8 percentage points (*p* < 0.001) (Figure 4).

## Discussion

This small single-centre project aimed to evaluate the implementation of a structured SOP, including a test dose protocol, and its impact on self-reported prescriber confidence. Prior to distribution of the SOP, most clinicians surveyed did not feel comfortable prescribing or distributing COX-2 inhibitors in the setting of AERD with under 15% stating they would use these treatments. This increased to over 80% following implementation, with over 90% of practitioners reporting the SOP was helpful in their decision making.

COX 2 inhibitors such as Celecoxib, were introduced in the early 1990s to address the side effects associated with NSAIDs (8). Several single- and double-blind placebo-controlled challenge studies have since demonstrated COX-2 inhibitors are a safe alternative for patients with AERD when given at therapeutic doses (3–5). In 2014 a meta-analysis of all controlled clinical trials showed no significant difference in respiratory symptoms, decrease in FEV1 (of 20% or greater), or nasal symptoms occurred taking COX-2 inhibitors (9). Despite this rare case reports have recounted COX-2 inhibitors inducing clinical symptoms such as urticaria, angioedema, rhinorrhoea, conjunctive erythema, bronchospasm, and dyspnoea in patients with severe AERD (10). Consequently, some experts have recommended to administer the first full dose of a COX-2 inhibitor in a health care setting to monitor for any adverse reactions (11). This precautionary has been incorporated into the SOP. Furthermore, studies advise initiating selective COX-2 inhibitors in patients with stable asthma only. In patients with uncontrolled asthma, it is advised to optimise asthma control with inhalers before administering selective COX-2 inhibitors. This aims to reduce the risk of adverse events in this vulnerable population (9). We have therefore integrated the European Respiratory Society (ERS) and American Thoracic Society (ATS) joint guidelines for the definition of uncontrolled asthma into our SOP to assist prescribers in assessing asthma control prior to COX-2 inhibitor initiation (12).

Managing pain in patients with AERD can be challenging with many patients relying on opioid analgesia to control perioperative pain. Codeine containing medications are not always tolerated in many patients due to various adverse effects. COX-2 inhibitors are an important alternative analgesic that can help achieve adequate post-operative pain control in AERD patients, both in relation to sinus surgery as well as other analgesia requirements. Despite this Morales et al. reported a lack of understanding of COX-2 inhibitors and their associated risks in AERD patients, noting they were often discouraged in clinical practice (9). NICE guidelines now recommend considering a COX-2 inhibitor in patients with reactions to NSAIDs (6). This study demonstrates that despite this evidence, there is still unease amongst healthcare professionals to use COX-2 inhibitors in asthmatic patients. This is not helped by regulatory agencies of COX-2 inhibitors still having a mandated warning for patients with NSAID hypersensitivity. The has often led to confusion about COX-2 inhibitors amongst patients and healthcare professionals (7). Our pre-SOP survey has further highlighted this lack of clarity, revealing significant uncertainty amongst prescribers regarding the safety and appropriate use of COX-2 inhibitors in AERD. These findings emphasise the need for a clear, evidence-based standard operating procedure (SOP) to support prescribers in making informed decisions. Following implementation of the SOP, our study was associated with a 68.8 percentage point increase in self-reported practitioner confidence in prescribing COX-2 inhibitors. This suggests a well-designed SOP could help provide clear prescribing pathways to reduce uncertainty and improve prescriber confidence around COX-2 inhibitors, ultimately enhancing post operative pain outcomes for this complex population of patients.

However, this small, self-selected, single centre project does have several limitations. Firstly, the sample size was relatively small, particularly in the follow up phase, which may limit the external validity of our findings. Secondly, although the pre- and post-SOP surveys were distributed to the same group of prescribers, the two respondent sample groups were not identical in composition, with differing proportions of professional groups responding to each survey. This represents an important limitation and a potential source of selection bias, as variation in professional role and specialty may influence prescribing confidence. Notably, the proportion of ENT consultant respondents was higher in the post-SOP survey, given their specialist expertise, they are likely to have greater baseline familiarity and confidence in prescribing COX-2 inhibitors in patients with AERD. Therefore, the observed increase in prescribing confidence should be interpreted with caution. It may reflect differences in respondent sample rather than a true effect of the SOP intervention. We aim to continue collecting data from prescribers who read the SOP to reduce this bias. Additionally, the project exclusively relies on prescribers self-reported confidence levels rather than objective measures and does not demonstrate changes in prescribing behaviours or patient outcomes. Further evaluation of COX-2 inhibitor prescribing rates before and after SOP implementation would help address this gap. Despite these limitations, the findings suggest that simple and concise educational interventions may play a valuable role in improving prescribing safety and clinician confidence in complex patient populations.

In summary, the burden of illness associated with AERD is significant. Although COX-2 inhibitors are recognised as a safe alternative for analgesia in AERD patients, clinician uncertainty persists. Our project highlights a lack of confidence in prescribing COX-2 inhibitors for patients with AERD. Implementation of a structured SOP was associated with improved self-reported prescribing confidence; however, this increase should be interpreted with caution due to differences in the surveyed populations between pre- and post-SOP surveys. Additionally, the effects on prescribing behaviour and patient outcomes remain to be established. Despite these limitations, over 90% of prescribers found the SOP beneficial. These findings highlight the potential value of clear prescribing guidelines, which may help bridge the knowledge gaps around COX-2 inhibitors and optimize pain management in this challenging patient population.

## Data Availability

The raw data supporting the conclusions of this article will be made available by the authors, without undue reservation.
