# Supplementary material for: Improving prescriber confidence in COX-2 inhibitor use for aspirin-exacerbated respiratory disease: impact of a standard operating procedure
Source: Front Allergy. 2026 Jun 22;7:1854011. doi: 10.3389/falgy.2026.1854011 (PMC13333619; doi:10.3389/falgy.2026.1854011)
Supplement: Supplementary file 1 [file Datasheet1.pdf]

Standard Operating Procedure

Use of COX-2 inhibitors in Peri-operative settings in patients with Aspirin (NSAID) exacerbated respiratory disease

<u>Use of COX-2 inhibitors in Peri-operative settings in patients with Aspirin (NSAID) exacerbated respiratory disease</u>

Quick Reference Guide

Pre-op ENT

- Confirm intolerance of AERD with patient
- Establish that asthma is well controlled
- Discuss use of COX-2 inhibitor with pateient during consent process

Peri-operative Aneasthetics

- Test dose of initially 40mg of paracoxib to be given IV intraoperatively (body weight up to 50kg - initially 20mg)
- Patient monitored for reaction
- Recovery staff informed and asked to monitor

Post-operative ENT

- Discharge patient with celecoxib 100mg bd
- Document on prescription that patient has tolerated test dose
- Document in discharge letter that patient may take COX-2 inhibitors without reaction

Introduction

Aspirin exacerbated respiratory disease (AERD)/Samter's triad is an inflammatory disease of the upper and lower airways characterised by eosinophilic chronic rhinosinusitis with nasal polyposis and asthma (1) as well as intolerance to non-selective cyclooxygenase (COX) inhibitors such as aspirin and NSAIDs (1, 2). The COX enzyme has two known isoforms COX-1 and COX-2. Pharmaceutical inhibition of COX-2 can help to relieve pain and inflammation, however non-selective COX inhibitors inhibit varying amounts of both isoforms (2). The mechanism of this hypersensitivity reaction in asthmatics is complex. Trials to assess the cross reactivity of COX 2 inhibitors in AERD patients have strongly supported the inhibition of COX 1 as the essential initiator of this inflammatory process (3-5).

COX 2 inhibitors such as Celecoxib, were introduced in the early 1990s to address the side effects associated with NSAIDs (6). Several single- and double-blind placebo-controlled challenge studies have since demonstrated COX-2 inhibitors are a safe alternative for patients with AERD when given at therapeutic doses (3-5). In 2014 a meta-analysis of all controlled clinical trials reported no significant difference in respiratory symptoms, decrease in FEV1 (of 20% or greater), or nasal symptoms occurred taking COX-2 inhibitors (7). Very rarely COX 2 inhibitors have induced clinical symptoms such as urticaria, angioedema, rhinorrhoea, conjunctive erythema, bronchospasm, and dyspnoea in patients with severe AERD (8).

Scope of Guideline

It is reported 5.4 million people in the UK suffer from asthma, with an estimated 5% to 15% of these patients having AERD (9, 10). In asthmatics with additional nasal polyposis the prevalence may be as high as 30% (10). The symptoms of AERD are difficult to treat, often not responding to standard medical interventions (1). Surgical intervention for nasal polyps is often required, with a high rate of polyp recurrence and revision surgery (10). It is important to note, up to 15% of patients are unaware they have NSAID hypersensitivity and will only be diagnosed via direct challenge (10).

Managing pain in patients with AERD can be challenging with many patients relying on opioid analgesia to control perioperative pain. Codeine containing medications are not always tolerated in many patients due to various adverse effects . COX-2 inhibitors are an important alternative analgesic that can help achieve adequate post operative pain control in AERD patients. Despite this Morales et al reported a lack of understanding of COX-2 inhibitors and their associated risks in AERD patients, noting they were often discouraged in clinical practice (7).

NICE guidelines now recommend considering a selective COX-2 inhibitor in patients with reactions to NSAIDs (9). However, there is still unease among healthcare professionals to use COX-2 inhibitors in asthmatic patients. This is not helped by regulatory agencies of COX-2 inhibitors still having a mandated warning for patients with NSAID hypersensitivity. The has often led to confusion about COX-2 inhibitors among patients and healthcare professionals (10).

The aim of this guideline is to address the confusion surrounding the use of COX2 inhibitors in patients with AERD and provide guidance as to their use in the perioperative setting.

Intervention

These medications will be prescribed and administered by Anaesthetists and ENT Surgeons peri-operatively. They will be dispensed for patients to take home by pharmacists within the hospital.

It is important when prescribing selective COX-2 inhibitors to discuss the benefits and risks of the medication, including the low risk of drug allergy (9).

It is also important to establish how well controlled the patient's asthma is, when prescribing COX-2 inhibitors (see figure 1) (7).

Populations

Groups that will be covered

Adults (aged 18 or over) with AERD undergoing a procedure at GSTT

Groups that will not be covered

Children (younger than 17) suffering from AERD

Healthcare setting

A Secondary and Tertiary health care facility (GSTT)

Contraindications

Studies have advised only initiating low doses of selective COX-2 inhibitors in patients with stable asthma. In patients with uncontrolled asthma, it is advised to optimise asthma control with inhalers before administering selective COX-2 inhibitors. This will help to reduce the risk of adverse events in this population (7).

Dosage and Administration

- Following NICE guidelines, it would be advised to introduce the lowest dose of a selective COX-2 inhibitor with only one dose on the first day (9).
- **A test dose of IV Paracoxib 40mg (body weight up to 50kg - initially 20mg) should be given during their surgical admission to monitor for any reaction and give patients confidence to use the medication after discharge** (10).

Results from previous trials recommended therapeutic doses of either 100mg or 200mg of Celecoxib were well tolerated in asthmatic patients with proven AERD (5).

Post operatively patients should be discharged with celecoxib 100mg BD. It should be well documented on the prescription that the patient has tolerated a test dose of IV Paracoxib. It is also important to document in the discharge letter that the patient may take COX-2 inhibitors without reaction

References

1. Lee R, Stevenson D. Aspirin-Exacerbated Respiratory Disease: Evaluation and Management. Allergy, Asthma and Immunology Research. 2010;3(1).
2. Qureshi O, Dua A. COX Inhibitors. StatPearls Publishing; 2024.
3. Stevenson D, Simon R. Lack of cross-reactivity between rofecoxib and aspirin in aspirin-sensitive patients with asthma. The Journal of Allergy and Clinical Immunology. 2001;108(1).
4. Gyllfors P, Bochenek G, Overholt J, Drupka D, Kumlin M, Sheller J, et al. Biochemical and clinical evidence that aspirin-intolerant asthmatic subjects tolerate the cyclooxygenase 2-selective analgetic drug celecoxib. The Journal of Allergy and Clinical Immunology. 2003;111(5).
5. Woessner K, Simon R, Stevenson D. The safety of celecoxib in patients with aspirin-sensitive asthma. Arthritis and Rheumatism. 2002;46(8).
6. Hawkey C. COX-2 Chronology. Gut. 2005;54(11).
7. Morales DR, Jackson C, Lipworth BJ, Donnan PT, Bruce G, Santiago VH. Safety risks for patients with aspirin-exacerbated respiratory disease after acute exposure to selective nonsteroidal anti-inflammatory drugs and COX-2 inhibitors: Meta-analysis of controlled clinical trials. Journal of Allergy and Clinical Immunology. 2014;134(1).
8. Umemoto JT, N. Nogi, S. Iwata, K.Oshikata, C. Tatsuno, S. Sekiya, K. Tsuburai, T. Akiyama, K. Selective cyclooxygenase-2 inhibitor cross-reactivity in aspirin-exacerbated respiratory disease. Allergy and Asthma Proceedings. 2012;32.
9. NICE. Drug allergy: diagnosis and management. NICE; 2014.
10. Haque RW, Andrew. Jackson, David. Hopkins, Claire. Clinical evaluation and diagnosis of aspirin-exacerbated respiratory disease. Journal of Allergy and Clinical Immunology. 2021;148:283-91.
